# Effect of Preheating Parameters on Extrusion Welding of High-Density Polyethylene Materials

**DOI:** 10.3390/polym16212992

**Published:** 2024-10-25

**Authors:** Chungwoo Lee, Suseong Woo, Sooyeon Kwon, Jisun Kim

**Affiliations:** 1 Purpose Built Mobility Group, Korea Institute of Industrial Technology, Gwangju 61012, Republic of Korea; leecw@kitech.re.kr (C.L.); mercury@kitech.re.kr (S.W.); 2 Department of Metallurgical Engineering, Jeonbuk National University, Jeonju-si 54896, Republic of Korea; 3 Fishing Vessel Safety Research Team, Korea Maritime Transportation Safety Authority, Sejong-si 30100, Republic of Korea

**Keywords:** high-density polyethylene (HDPE), extrusion welding, preheating conditions, performance testing, joint efficiency

## Abstract

High-density polyethylene (HDPE) has emerged as a promising alternative to fiber-reinforced plastic (FRP) for small vessel manufacturing due to its durability, chemical resistance, lightweight properties, and recyclability. However, while thermoplastic polymers like HDPE have been extensively used in gas and water pipelines, their application in large, complex marine structures remains underexplored, particularly in terms of joining methods. Existing techniques, such as ultrasonic welding, laser welding, and friction stir welding, are unsuitable for large-scale HDPE components, where extrusion welding is more viable. This study focuses on evaluating the impact of key process parameters, such as the preheating temperature, hot air movement speed, and nozzle distance, on the welding performance of HDPE. By analyzing the influence of these variables on heat distribution during the extrusion welding process, we aim to conduct basic research to derive optimal conditions for achieving strong and reliable joints. The results highlight the critical importance of a uniform temperature distribution in preventing defects such as excessive melting or thermal degradation, which could compromise weld integrity. This research provides valuable insights into improving HDPE joining techniques, contributing to its broader adoption in the marine and manufacturing industries.

## 1. Introduction

FRP (Fiber-Reinforced Plastic) has been used in small hulls due to its high strength and rigidity, but concerns such as fire vulnerability and environmental pollution upon disposal have been raised [1,2,3,4]. In response to the increasing global demand for environmentally friendly materials to replace FRP, HDPE has emerged as a promising alternative for small hull manufacturing [5,6,7,8,9]. Given its excellent durability, chemical resistance, lightweight nature, and recyclability, HDPE is expected to be highly useful in marine environments where high humidity and corrosion are prevalent [10,11,12].

Currently, the joining of thermoplastic polymers like HDPE has primarily been used for gas and water pipelines [13,14,15,16]. Because gas and water pipelines have standardized sizes, research has focused on joining methods such as ultrasonic welding, laser welding, friction stir welding, and heat fusion [17,18,19,20,21,22]. However, to utilize HDPE, which has excellent performance, in complexly shaped and large-scale hulls and marine structures, appropriate welding and joining technologies are needed to ensure structural integrity. Since fusion welding is practically difficult to use, it is necessary to explore joining methods using extrusion-based equipment [23,24,25].

However, the joining of HDPE using extrusion methods is still underdeveloped compared to traditional welding techniques used with metal materials. As a result, the quality of joining technology for HDPE has not been adequately established, and the joints often lack sufficient strength and quality. This presents a significant challenge in developing suitable joining techniques for hulls and marine structures [26,27,28]. Consequently, the current practice heavily relies on the skills of field operators performing HDPE joining as it depends on the expertise of experienced workers [29,30].

HDPE materials used in large-scale industries must be joined using extrusion methods; however, research analyzing the relationship between specific factors and joining performance for extrusion methods is notably lacking [31]. Due to the relatively sparse research on joining related to extrusion methods, factors influencing joining performance have been derived based on prior studies using other joining methods for HDPE materials. It has been confirmed that various process variables, such as temperature and pressure applied during the melting process, affect the joining performance of HDPE materials [32,33,34,35,36,37,38,39].

Thermoplastic polymers like HDPE exhibit variations in joining performance depending on processing conditions. This study investigates the impact of process conditions on the joining performance in extrusion-based joining processes, which are essential for complexly shaped and large-scale hulls and marine structures. This research aims to review how process conditions affect joining performance and conduct fundamental studies to achieve superior joining performance.

In particular, due to the low thermal conductivity and low melting temperature characteristics [40], preheating the surface of the base material exposed to heat during the joining process is considered to have a major impact on the joining performance of HDPE. Therefore, this study analyzes how parameters such as hot air height, hot air movement speed, and the distance from the center of the hot air flow affect the thermal distribution across various areas of the HDPE-based material. Finally, by evaluating the shear stress of specimens joined under different preheating conditions, this study provides a comprehensive discussion of the differences in thermal distribution across the substrate during preheating and how these variations influence joining performance. The findings underscore the importance of temperature uniformity in preventing defects such as excessive melting or thermal damage that can degrade joining performance. This research aims to contribute to overcoming the current limitations of polymer joining and advancing the development of HDPE materials, which have significant potential applications in the marine and extensive manufacturing industries.

## 2. Materials and Methods

### 2.1. Base Material Properties

To analyze the trend in preheating temperature variations in HDPE base material according to joining process variables, HDPE (EcoMarine (Busan, Republic of Korea), HDPE) with dimensions of 120 mm (L) × 240 mm (W) × 10 mm (H) was used as the base material. In the experiments, one side of the HDPE base material was machined with a v-groove modification, and two machined HDPE base materials were arranged in a butt joint configuration for use. Table 1 shows the mechanical properties of the HDPE base material used in the experiments.

### 2.2. Welding Equipment and Parameters

**Figure 1:**
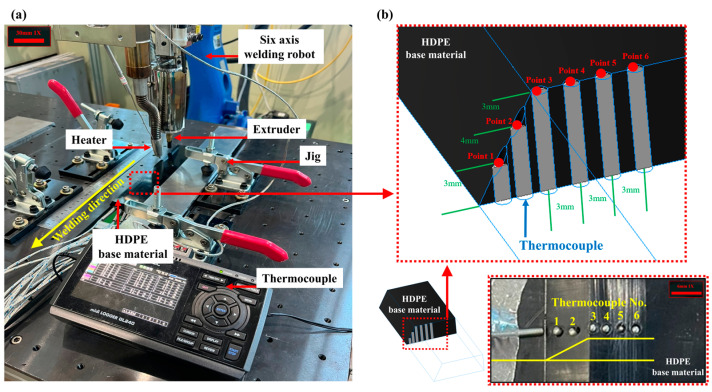
Experimental setup: (**a**) experimental equipment; (**b**) thermocouple positions.

To ensure consistent welding process parameters, an extrusion-type extruder (Sinwoo (Seoul, Republic of Korea), D4) capable of wire-based welding was attached to a six-axis articulated robot (Yaskawa (Kitakyushu, Japan), MH6). A hot air blower was mounted in front of the welding equipment’s travel direction to preheat the HDPE base material by applying hot air at a constant temperature, with the blower maintaining a 90° working angle relative to the base material. To measure the preheating temperature based on the thermocouple positions within the base material, a data logger (Graphtec (Yokohama, Japan), GL240) equipped with thermocouples was used. A custom clamp-type jig was employed to fix the positions of the thermocouples and the base material. During the preheating experiments simulating the welding process, temperature changes over time were measured at specific points where the thermocouples were attached to the base material. Figure 1 shows the configuration of the experimental equipment used in this study and the thermocouple attachment points.

### 2.3. Experimental Design

**Figure 2:**
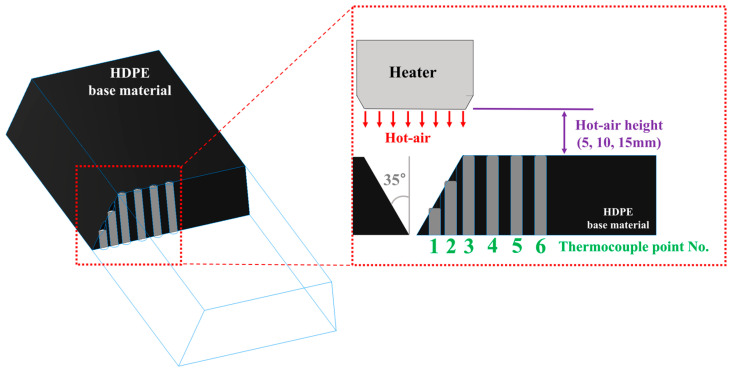
Schematic diagram of welding process parameters.

An experimental plan was developed to analyze the changes in preheating temperature of the HDPE base material based on hot air movement speed and hot air height, which could affect the preheating temperature of the base material, with the hot air temperature being maintained at 258.2 ± 3.5 °C. Table 2 presents the experimental design for analyzing the preheating temperature changes in the HDPE base material according to the welding process parameters, while Figure 2 shows a schematic diagram of the welding process parameters. The hot air height was measured based on the position where the welding equipment, maintaining a 90° working angle relative to the HDPE base material, makes contact and starts applying pressure in the actual HDPE welding process. The hot air movement speed was calculated as the joining speed that can form a good bead while filling the entire volume of the v-groove improvement processed at 35° in the butt joint based on the material throughput specifications of each stage of the used joining equipment. Since the hot air blower is attached to the welding equipment, the welding speed was used as the hot air movement speed in the experimental design.

### 2.4. Method of Mechanical Property Evaluation

**Figure 3:**
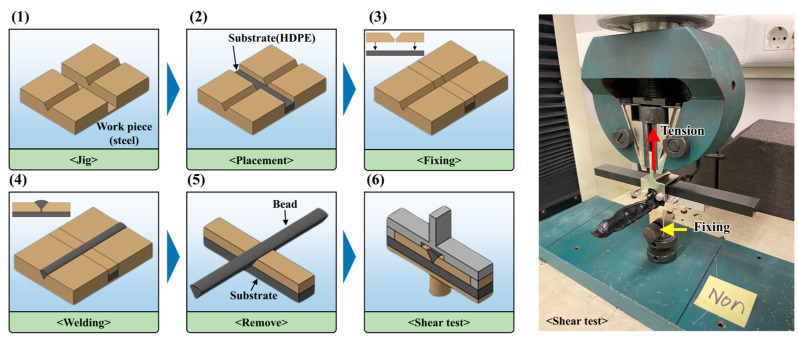
Self-made shear jig and shear test schematic.

To analyze and discuss the changes in the preheating temperature of the HDPE base material according to the welding process parameters, we compared joints without preheating and those preheated with hot air. The joining experiment used a 4 mm diameter HDPE wire (Röchling-ReLoop (Worms, Germany), HDPE). It has a melt index of 0.12 g/min, a crystallization temperature of 120 °C, and a melting point of 130 °C. To prevent preheating temperature differences due to varying geometries, the Bead-on-Plate (BoP) method was employed. The welding experiment was conducted under consistent conditions of a 5 mm preheating height, a 1.8 kg/h material throughput, and a 35 cm/min hot air movement speed. To analyze the effect of preheating on the joints, a shear test was performed. The shear test was conducted using a universal testing machine (Tinius Olsen(Redhill, UK), H10KS), where the base material was fixed with a jig and the welded bead was pulled in the opposite direction of gravity at a speed of 5 mm/min. A self-made shear jig was used for the shear test, and a schematic diagram is shown in Figure 3. The shear test was performed with 5 repetitions, resulting in an error of at least 2.3%

## 3. Preheating Experiment Results

**Figure 4:**
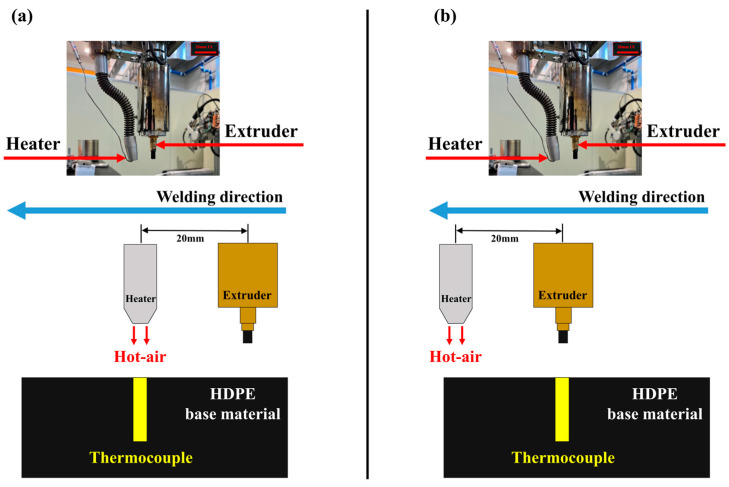
A schematic diagram of the process of when the hot air blower and wire extrusion unit are positioned at the thermocouple: (**a**) the hot air blower; (**b**) the extrusion unit.

To analyze the results of the preheating temperature change experiments at different points within the base material, which were conducted according to the experimental plan, the temperature data measured over time were compared. Specifically, the temperature recorded when the hot air blower and wire extrusion unit were positioned over the thermocouples attached to the base material as well as the maximum temperature at each point were analyzed. Figure 4 provides a schematic illustration of when the hot air blower and wire extrusion unit are positioned over the thermocouples attached to the base material. Since the hot air blower is located in front of the wire extrusion unit in the welding direction, the hot air blower passes over the thermocouple position first, followed by the wire extrusion unit. The temperature at the point when the wire extrusion unit reaches the thermocouple is considered the actual preheating temperature relevant for welding, making it the key temperature for analysis. Due to the different positions of the hot air blower and the wire extrusion unit, the preheating temperature of the base material at the thermocouple positions varies depending on which part of the welding equipment is located at those positions.

### 3.1. Preheating Temperature Trends

**Figure 5:**
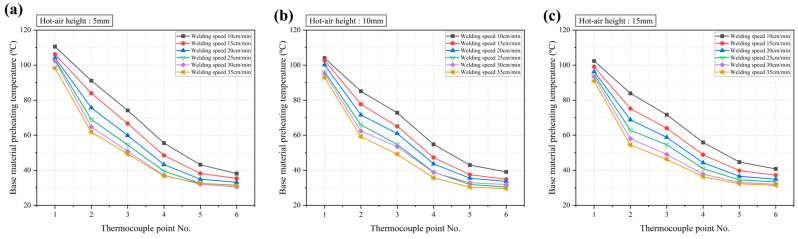
Preheating temperature at thermocouple positions for different hot air heights: (**a**) 5 mm, (**b**) 10 mm, and (**c**) 15 mm.

Since the preheating temperature of the base material when the wire extrusion unit of the welding equipment is positioned at the thermocouple attached to the base material is the actual preheating temperature during welding, this temperature is referred to as the preheating temperature at the moment of welding. The results were analyzed based on this definition. Figure 5 shows the preheating temperature measurements at different points within the base material at the moment of welding. Figure 5a–c confirm that as the hot air movement speed increased, the preheating temperature of the base material decreased linearly regardless of the thermocouple position or hot air height. Additionally, under the same conditions, the preheating temperature decreased as the hot air height increased, and as the thermocouple points were located further from the center of the base material, the preheating temperature also decreased. Among the six position points, positions 1 to 4 showed clear trends in temperature increase and decrease, while positions 5 and 6 exhibited less deviation compared to positions 1 to 4.

**Figure 6:**
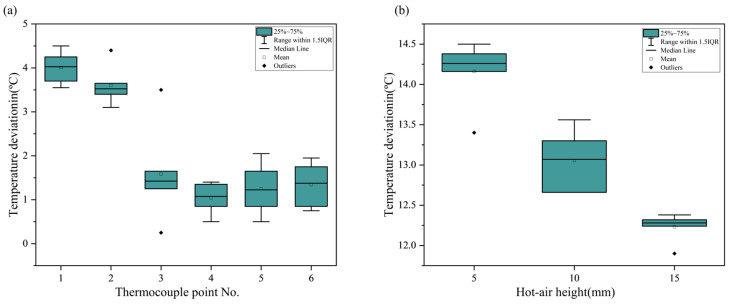
Temperature deviation by thermocouple position: (**a**) Deviation by hot air height. (**b**) Deviation by distance from center of hot air flow.

Under the same hot air conditions, despite the thermocouple at position 3 being located lower than that at position 1 due to the v-groove geometry, the maximum temperature was observed at position 1 rather than position 3. This suggests that the distance from the center of the hot air flow has a greater effect on the preheating temperature than the height of the hot air. This result is also evident in Figure 6, which shows that the temperature deviation based on the thermocouple position was larger than the deviation due to the hot air height. As shown in Figure 6a, the difference in temperature due to the hot air height was small, with a maximum difference of 4.5 °C. The temperature deviation at positions 3 to 6 was about 1 °C and showed similar results, while positions 1 and 2 exhibited relatively higher temperature deviations. These results indicate that the influence of hot air height is concentrated at positions 1 and 2, with less effect on positions 3 to 6. From the results in Figure 6b, it is confirmed that the temperature deviation decreased as the hot air height increased. This is believed to be due to the reduction in the temperature of the hot air as the distance from the base material’s surface increased, leading to less direct contact with the surface.

### 3.2. Comparison of Preheating Temperature

**Figure 7:**
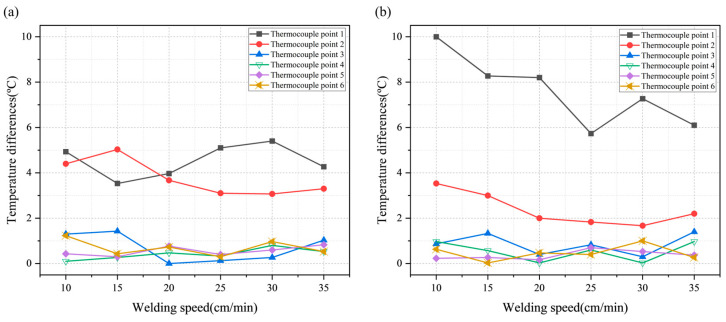
Differences in preheating temperature based on hot air movement speed and hot air height: (**a**) preheating temperature at moment of welding; (**b**) maximum preheating temperature.

The difference in preheating temperature at various points within the base material, between the maximum preheating temperature and the preheating temperature at the moment of welding, was analyzed based on the hot air movement speed. Figure 7 shows the differences in preheating temperature according to the hot air movement speed and hot air height, both at the moment of welding and at the maximum preheating temperature. As shown in Figure 7a, the temperature difference at positions 3–6 at the moment of welding was within 2 °C, while positions 1 and 2 showed a relatively higher temperature difference compared to positions 3–6. Additionally, there was no evident trend in preheating temperature based on the hot air movement speed. These results suggest that when the hot air height changes, the temperature variation is more pronounced in the groove shape than on the upper surface of the base material. From the results of the preheating temperature difference, it was confirmed that the upper surface of the base material is not significantly affected by changes in the hot air movement speed or hot air height. Instead, the hot air movement speed and height mainly influence the preheating of the surface closer to the center of the hot air stream within the groove shape. Figure 7a,b show a clear temperature difference at positions 1 and 2. The maximum temperature at position 1 exhibited a larger difference from the preheating temperature at the moment of welding than at positions 1–6. Position 1, which is most affected by hot air, showed a rapid increase in preheating temperature when the hot air passed over the thermocouple. However, due to the low thermal conductivity of the HDPE material, the surface heat did not penetrate into the material but was quickly dissipated outward.

### 3.3. Preheating Results over Time

**Figure 8:**
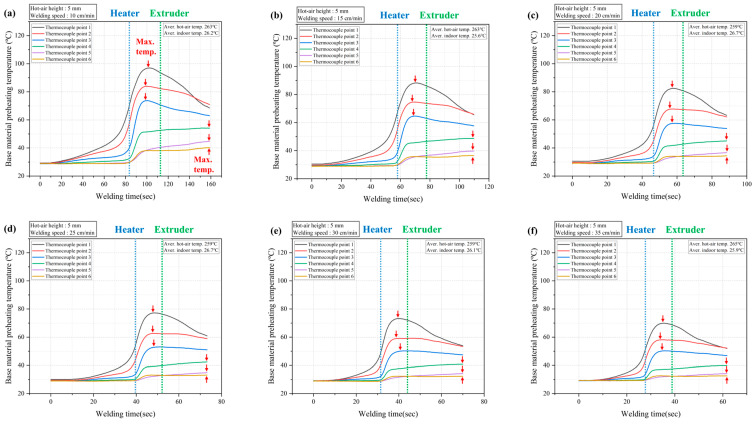
Changes in preheating temperature over time: welding speeds of (**a**) 10 cm/min, (**b**) 15 cm/min, (**c**) 20 cm/min, (**d**) 25 cm/min, (**e**) 30 cm/min, and (**f**) 35 cm/min.

Figure 8 shows the changes in preheating temperatures at the thermocouple points over time. No significant trends related to the hot air movement speed were observed. Due to the structure of the welding equipment, where the hot air blower is positioned ahead of the wire extruder, and the limited range affected by the hot air, the time it took to reach the maximum temperature varied depending on the thermocouple location points. At positions 1 to 3, which are located on the v-groove prepared on the base material and are directly impacted by the hot air, the maximum temperature was reached after the hot air blower passed but before the wire extruder arrived at the thermocouple. On the other hand, at positions 4 to 6, which were less directly exposed to the hot air on the surface of the base material, the maximum temperature was reached over time after the wire extruder had passed. This result suggests that the preheating temperature at points 4 to 6, which were not directly affected by the hot air, increased due to the overall heating effect on the base material caused by the hot air directly impacting positions 1 to 2.

## 4. Mechanical Properties Evaluation and Discussion

### 4.1. Shear Stress

**Figure 9:**
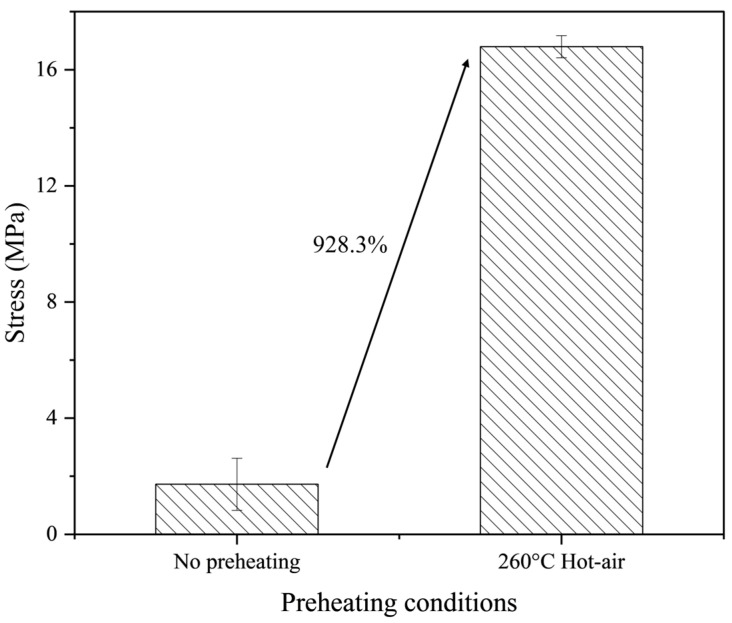
Shear stress measurement results based on preheating.

In the welding process of the HDPE material, the trend in preheating temperature changes at different surface points of the base material was examined. To understand how the difference in preheating at various points affects weldability, the shear stress of a joint without preheating and a joint preheated using hot air on the base material was compared. Figure 9 presents the shear stress measurement results based on preheating. A clear difference in shear stress was observed between the preheated and non-preheated joints. The joint preheated at a hot air temperature of 260 °C exhibited a shear stress value over nine times higher than the non-preheated joint. These results suggest that preheating the base material significantly influences the welding performance of HDPE material.

**Figure 10:**
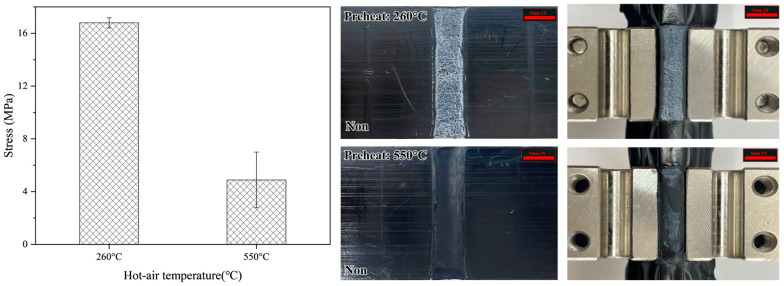
Shear stress results of HDPE joints based on hot air temperature conditions.

Having confirmed that preheating the surface of the base material impacts the welding performance, a comparison was made to analyze the difference in shear stress based on different preheating temperatures. The shear stress results of a joint preheated at a hot air temperature of 550 °C were compared with those of a joint preheated at a hot air temperature of 260 °C. Figure 10 shows the shear stress results for joints preheated at the two hot air temperature conditions of 260 °C and 550 °C. While the joint preheated at a hot air temperature of 260 °C showed a higher shear strength than the non-preheated joint, it was found that the shear stress of the joint preheated at a hot air temperature of 550 °C was lower than that of the joint preheated at a hot air temperature of 260 °C. This result indicates that excessive melting in the welding process of HDPE material can reduce the joint strength. The reduction in shear stress is likely due to the unraveling of numerous polymer chains as a result of excessive melting, as polymer chains have different lengths and therefore unravel at different temperatures [41,42,43,44]. Thus, selecting appropriate preheating temperature is essential for the welding process of HDPE materials.

### 4.2. Discussion and Considerations

**Figure 11:**
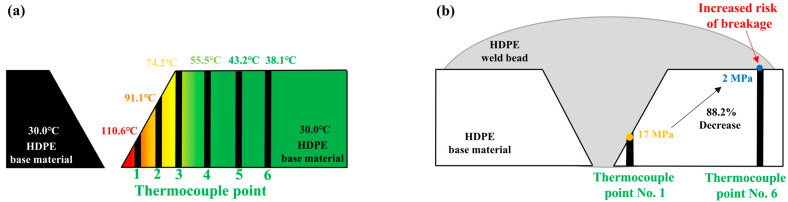
A schematic diagram of the welding process for HDPE material with an improved v-groove shape: (**a**) the preheating temperature distribution; (**b**) issues in the joint.

Figure 11 shows a schematic diagram of the preheating temperature distribution and welding results in the butt joint process of HDPE material with an improved v-groove shape. As shown in Figure 11a, only the temperature increase effect, resulting from the overall preheating of the base material, was observed on the upper surface of the base material compared to the v-groove improvement area where the hot air is concentrated. This indicates that a temperature difference occurs within the butt joint of the HDPE material with an improved v-groove shape. The results in Section 4.1 show that the preheated joint exhibited superior shear stress compared to the non-preheated joint, and excessive melting was identified as a factor reducing joint strength. Therefore, differences in welding performance may arise due to the variation in the preheating temperature across different locations of the base material.

HDPE material has a lower thermal conductivity compared to metals, so the preheating effect from the hot air does not quickly spread throughout the base material. As a result, temperature differences appear at different locations within the base material that are not directly exposed to the hot air. As shown in the preheating results in Figure 5 and Figure 11b, the surface of the base material, where the preheating temperature is lower compared to other locations, may result in a lower welding performance compared to the preheated joint, potentially leading to joint failure. Therefore, minimizing the temperature variation across the joint is critical for achieving superior weld performance. Additionally, a solution is needed to increase the preheating temperature on the base material surface, where the preheating effect is insufficient, while preventing excessive melting in the v-groove area. In the future, a further analysis of the joint performance based on the pressure conditions of the welding equipment and the surface roughness of the base material will be necessary.

## 5. Conclusions

In this study, changes in the base material under various preheating process conditions during the welding process of the HDPE material were experimentally investigated. Based on the impact of process conditions on the preheating results of the base material, the following conclusions were drawn from the analysis of the experiment results aimed at improving joint performance.

Preheating experiments were conducted to examine the effects of the hot air height, hot air movement speed, and distance from the hot air center on the preheating of the base material. Both the hot air height and hot air movement speed were found to have an inverse relationship with the preheating temperature of the HDPE base material.

The distance from the hot air center, measured perpendicularly to the welding path, showed a decrease in preheating temperature as the distance increased. A comparison of the temperature differences based on the hot air height and distance from the center revealed that the distance from the hot air center had a greater influence on the preheating temperature change in the base material than the height of the hot air.

In the butt welding of HDPE material with a v-groove modification, the surface of the base material that did not undergo direct exposure to the hot air did not exhibit a clear trend under different hot air process conditions. It was found that the surface temperature only increased due to the overall preheating of the base material, which is attributed to the low thermal conductivity of HDPE material.

The joints of preheated HDPE base material exhibited superior shear strength compared to non-preheated joints. However, excessive melting caused by higher preheating temperatures resulted in a reduced joint strength. Therefore, it is crucial to select appropriate preheating temperature conditions within a certain range during the welding process of HDPE material.

Since variations in the surface preheating temperature of the base material can cause damage, minimizing the temperature difference within the joint while avoiding excessive melting is considered the most crucial aspect of enhancing joint performance. To achieve optimal joint performance, it is necessary to modify the shape of the hot air blower and improve the design of the weld zone.

Through this study, it was confirmed that preheating process conditions are a key variable that determines joint performance in the welding process of HDPE material. The results are expected to contribute to the development of an optimal welding performance for HDPE material.

## Figures and Tables

**Table 1:** Mechanical properties of HDPE base material (EcoMarine (Republic of Korea), HDPE).

Variable	Level
Density (g/cm^3^)	0.95
Elongation percentage (%)	261
Izod impact value (J/m)	631
Tensile strength (MPa)	29.2
Flexural modulus (MPa)	1340
Melt index (g/min)	0.089
Crystallization temperature (°C)	120
Melting point (°C)	130

**Table 2:** Experimental plan.

Test No.	Hot Air Movement Speed (cm/min)	Hot Air Height (mm)
1	10	5
2	15	5
3	20	5
4	25	5
5	30	5
6	35	5
7	10	10
8	15	10
9	20	10
10	25	10
11	30	10
12	35	10
13	10	15
14	15	15
15	20	15
16	25	15
17	30	15
18	35	15

## Data Availability

The original contributions presented in this study are included in the article; further inquiries can be directed to the corresponding authors.
